# Metabolite cross-feeding enables concomitant catabolism of chlorinated methanes and chlorinated ethenes in synthetic microbial assemblies

**DOI:** 10.1093/ismejo/wrae090

**Published:** 2024-05-31

**Authors:** Gao Chen, Yi Yang, Jun Yan, Frank E Löffler

**Affiliations:** Department of Civil and Environmental Engineering, University of Tennessee, Knoxville, TN 37996, United States; Key Laboratory of Pollution Ecology and Environmental Engineering, Institute of Applied Ecology, Chinese Academy of Sciences, Shenyang, Liaoning 110016, China; Key Laboratory of Pollution Ecology and Environmental Engineering, Institute of Applied Ecology, Chinese Academy of Sciences, Shenyang, Liaoning 110016, China; Department of Civil and Environmental Engineering, University of Tennessee, Knoxville, TN 37996, United States; Department of Microbiology, University of Tennessee, Knoxville, TN 37996, United States; Department of Biosystems Engineering and Soil Science, University of Tennessee, Knoxville, TN 37996, United States

**Keywords:** cross-feeding, co-cultures, synthetic ecology, chlorinated methanes, chlorinated ethenes, organohalide respiration, anaerobic food webs, natural halogen cycling, bioremediation

## Abstract

Isolate studies have been a cornerstone for unraveling metabolic pathways and phenotypical (functional) features. Biogeochemical processes in natural and engineered ecosystems are generally performed by more than a single microbe and often rely on mutualistic interactions. We demonstrate the rational bottom-up design of synthetic, interdependent co-cultures to achieve concomitant utilization of chlorinated methanes as electron donors and organohalogens as electron acceptors. Specialized anaerobes conserve energy from the catabolic conversion of chloromethane or dichloromethane to formate, H_2_, and acetate, compounds that the organohalide-respiring bacterium *Dehalogenimonas etheniformans* strain GP requires to utilize *cis*-1,2-dichloroethenene and vinyl chloride as electron acceptors. Organism-specific qPCR enumeration matched the growth of individual dechlorinators to the respective functional (i.e. dechlorination) traits. The metabolite cross-feeding in the synthetic (co-)cultures enables concomitant utilization of chlorinated methanes (i.e. chloromethane and dichloromethane) and chlorinated ethenes (i.e. *cis*-1,2-dichloroethenene and vinyl chloride) without the addition of an external electron donor (i.e. formate and H_2_). The findings illustrate that naturally occurring chlorinated C_1_ compounds can sustain anaerobic food webs, an observation with implications for the development of interdependent, mutualistic communities, the sustenance of microbial life in oligotrophic and energy-deprived environments, and the fate of chloromethane/dichloromethane and chlorinated electron acceptors (e.g. chlorinated ethenes) in pristine environments and commingled contaminant plumes.

## Introduction

In the environment, microorganisms usually exist within taxonomically and functionally diverse communities, where metabolically active cells constantly interact, e.g. by competing for nutrients or exchanging metabolites [1–3]. These microbe-microbe interactions can have beneficial, neutral, or harmful effects on members of the community and are key factors determining community function, response to perturbations, and resilience. For example, cross-feeding is an interaction between microorganisms in which molecules produced and released by one microbe (e.g. vitamins, amino acids, and metabolites) are utilized by another [4, 5]. Cross-feeding interactions can be unidirectional or bidirectional and represent common mutualistic relationships between microbes, providing a driving force for the maintenance of diversity and functional stability in microbial communities [5]. The lack of a thorough understanding of these microbial interactions impedes our ability to obtain many interesting organisms in axenic culture and develop a predictive understanding of biogeochemical processes. The knowledge-based design of synthetic communities with desirable functions by combining predefined axenic or enrichment cultures to achieve a desired function (i.e. synthetic ecology) has great promise but requires understanding of relevant microbe–microbe interactions [6–8]. Disentangling microbial interactions in natural ecosystems, such as the gut or soil, is challenging due to the intrinsic complexity of such relationships [9]. High-throughput sequencing provides insights into the taxonomic compositions of microbiomes and can reveal the metabolic potentials of community members; however, many challenges exist for microbial interaction network reconstruction and prediction of mutualistic relationships [10]. Detailed laboratory studies with synthetic microbial assemblies (i.e. synthetic microbiomes) can reveal important metabolic interactions underlying the functional attributes of a microbial community [11–13].

Many synthetic chemicals, such as chlorinated methanes and chlorinated ethenes, are notorious groundwater pollutants. By no means are chlorinated C_1_ and C_2_ compounds strictly synthetic xenobiotics (gr. xenos, foreign; bios, life; foreign to life), and natural systems produce a range of organohalogens. More than 5,000 halogenated chemicals, the majority of them chlorinated, are released into the environment by biological and abiotic (geo)chemical processes [14–16]. Chloromethane (CM) is formed during the degradation of organic matter [17–19], the senescence of plant material [20], and biomass burning [21], and is produced by marine macroalgae [22, 23]. CM is the most abundant organohalogen in the atmosphere, with annual global emissions from marine and terrestrial sources estimated to exceed 4,000 Gg [24–26]. The majority of dichloromethane (DCM) in the environment is of anthropogenic origin, with natural emissions from oceans, wetlands, mangroves, and volcanoes contributing ~63 Gg of DCM annually to the atmosphere [27–32]. In addition to CM and DCM, a diversity of organohalogens, including chlorinated ethenes, is produced through biotic and abiotic processes in marine and terrestrial environments [15, 16]. For example, vinyl chloride (VC) can abiotically form in soils through iron-catalyzed oxidation of humic acids and has been detected in volcanic emissions [33, 34].

In anoxic environments, DCM and CM can be fermented or mineralized by specialized anaerobes [35–37]. Axenic cultures of *Dehalobacterium formicoaceticum* strain DMC and strain CM, a new isolate obtained from sediments of an estuary, ferment DCM and CM, respectively, to formate, acetate, and inorganic chloride, while `*Candidatus* Dichloromethanomonas elyunquensis' mineralizes DCM to CO_2_, H_2_, and chloride under anoxic conditions [36–39]. Organohalide-respiring bacteria (OHRB) within the class *Dehalococcoidia* can utilize a variety of organohalogen compounds, including polychlorinated ethenes and VC, as growth-supporting electron acceptors [40–42]. To perform reductive dechlorination, *Dehalococcoides mccartyi* strains have a strict requirement for H_2_, whereas *Dehalogenimonas* spp. can also utilize formate as an electron donor with acetate serving as a carbon source.

To explore the poorly understood microbial interactions during the utilization of CM and DCM as sources of reducing equivalents (i.e. electron donors) and organohalogens (e.g. VC and cDCE) as electron acceptors, synthetic co-cultures were established. The co-cultures comprised an anaerobic CM- or DCM-degrading bacterium [36, 37] and the organohalide-respiring bacterium *D. etheniformans* strain GP, which is the first non-*Dehalococcoides* isolate capable of metabolic reductive dechlorination of trichloroethene (TCE), all dichloroethene isomers, and VC to ethene with formate or H_2_ as electron donor and acetate as carbon source [42, 43]. Metabolite cross-feeding supported growth of both population types and enabled concomitant utilization of the chlorinated methanes and the chlorinated ethenes as energy sources in the synthetic microbiomes without the need for an external electron donor (i.e. H_2_ and formate) and a carbon source (i.e. acetate). The new findings highlight the role of the natural chlorine cycle for the development of interdependent, mutualistic microbial communities and have implications for the microbial ecology in pristine and contaminated ecosystems.

## Materials and Methods

### Chemicals

DCM (purity ≥99.8%), CM (≥99.5%), *cis*-1,2-dichloroethene (cDCE, >96.0%), and ethene (≥99.5%) were purchased from Sigma-Aldrich (St Louis, MO, USA). Gaseous VC (purity >99%) was purchased from SynQuest Laboratories (Alachua, FL, USA). 2-Bromoethanesulfonate (BES, purity >98%) was purchased from Acros Organics (NJ, USA). All other chemicals used in this study were reagent-grade or of higher purity.

### Cultivation and synthetic microbiomes

Routine cultivation of the axenic cultures of *Dehalobacterium formicoaceticum* strain DMC, strain CM, and *D. etheniformans* strain GP, and consortium RM comprising the DCM-utilizing `*Ca. Dichloromethanomonas elyunquensis*' used 160-ml glass serum bottles containing 50 ml of bicarbonate-buffered (30 mM, pH 7.3) basal salt medium reduced with 0.2 mM Na_2_S, 0.2 mM l-cysteine, and 0.5 mM dithiothreitol (DTT) and amended with resazurin (0.25 mg L^−1^) as redox indicator [44]. The bottles were sealed with butyl rubber stoppers (Bellco Glass Inc., Vineland, NJ, USA) under a headspace of N_2_/CO_2_ (80/20, vol/vol). Strain CM received 3 ml of CM gas (122.6 μmol), and *Dehalobacterium formicoaceticum* strain DMC and consortium RM were provided with 5 μl of neat DCM (78.3 μmol) as the sole energy source. *D. etheniformans* strain GP was grown in basal salt medium amended with 2 mM formate, 2 ml VC (81.7 μmol, 1.6 mM nominal concentration), and 1 mM acetate as electron donor, electron acceptor, and carbon source, respectively. Strain CM was isolated with CM as the sole energy source from a salt-flat mud sample. The strict anaerobic bacterium ferments CM to formate and acetate in bicarbonate-buffered basal salt medium. The isolate’s 16S rRNA gene (GenBank accession number MW682303) shares 95.4% sequence similarity with *Sporobacter termitidis* strain SYR^T^ (Z49863.1) [45]; however, the classification of strain CM has to await its polyphasic characterization.

The co-culture batch experiments with strain CM and *D. etheniformans* strain GP were performed in 160-ml glass serum bottles containing 50 ml basal salt medium lacking reduced carbon compounds (e.g. formate and acetate). The reactor vessels were inoculated (3%, vol/vol, ~10^7^ cells ml^−1^) with strain CM and strain GP cultures. CM (~50 μmol) was amended as the substrate for strain CM and VC (~50 μmol) or cDCE (~40 μmol) was amended as the electron acceptor for *D. etheniformans* strain GP. The vessels received additional ~50 μmol CM when CM had been depleted. *Dehalobacterium formicoaceticum* strain DMC and *D. etheniformans* strain GP co-cultures received 3 μl DCM (47.0 μmol) and 1 ml VC (40.9 μmol). Upon DCM depletion, the cultures received additional 3- or 5-μl doses of DCM. All vessels were incubated in upright position at 30°C in the dark without agitation. The consumption of chlorinated substrates (i.e. DCM, CM, cDCE, and VC) and the formation of the reductive dechlorination product ethene were measures of activity. During growth, culture suspension samples (1 ml) were filtered onto 0.22 μm Durapore membranes (Millipore, Cork, Ireland), which were immediately stored at −80°C for subsequent DNA extraction and qPCR analysis. Culture suspension samples (1 ml) were also collected for acetate and formate measurement using HPLC. Co-culture suspension samples were regularly collected and visually examined with a Zeiss AX10 light microscope (Jena, Germany). Axenic cultures (*D. etheniformans*, *Dehalobacterium formicoaceticum*, and strain CM) and consortium RM amended with a chlorinated methane (CM or DCM) and a chlorinated ethene (VC or cDCE) served as controls. Incubation vessels receiving autoclaved inocula served as abiotic controls.

### Quantitative polymerase chain reaction (qPCR)

Organism-specific qPCR assays each targeting the 16S rRNA gene of strain CM, *Dehalobacterium formicoaceticum* strain DMC, and `*Ca*. *Dichloromethanomonas elyunquensis*' strain RM, and the VC reductive dehalogenase gene (*cerA*) of *D. etheniformans* strain GP were used to enumerate cell numbers and monitor the growth of the respective populations in the co-cultures. The genomes of the bacteria included in this study harbor a single 16S rRNA gene, except *Dehalobacterium formicoaceticum* strain DMC, with four copies per genome [46–48]. DNA was extracted using the DNeasy PowerLyzer PowerSoil DNA isolation kit (Qiagen, Hilden, Germany) following the manufacturer’s protocol. qPCR primers and probes are listed in Table 1 or have been reported [38, 42]. The qPCR analysis followed published protocols and was conducted using an ABI ViiA7 real-time PCR system (Life Technologies). Briefly, every 20-μl reaction mixture contained 10 μl of 2X Taqman Universal PCR Master Mix (Applied Biosystems, Carlsbad, CA, USA), 2 μl of DNA template (DNA concentrations ranging between 20 and 100 ng μl^−1^), and forward and reverse primers and probes at final concentrations of 300 nM each. The PCR thermal cycling protocol was as follows: 50°C for 2 min, then at 95°C for 10 min, followed by 40 cycles of denaturation at 95°C for 15 s, and annealing and extension at 60°C for 1 min. Calibration curves used serial 10-fold dilutions of the synthetic linear DNA fragment LDFA1 (1500 bp, GeneArt Strings DNA Fragments; Invitrogen, CA, USA) comprising partial gene fragments of the 16S rRNA genes of strain CM, *Dehalobacterium formicoaceticum* strain DMC, and the VC reductive dehalogenase gene *cerA* with the respective primer and probe binding sites. For 'Ca. *Dichloromethanomonas elyunquensis*' strain RM, calibration curves used serial 10-fold dilutions of plasmid DNA carrying a cloned 16S rRNA gene of the DCM-degrading strain RM (pCR 2.1-TOPO vector; Invitrogen, Carlsbad, CA, USA) and spanned a concentration range from 3.44 × 10^8^ to 34.4 target gene copies per assay tube [38].

**Table 1:** Primers and probes used for qPCR assays specifically targeting 16S rRNA genes of strain CM and *Dehalobacterium formicoaceticum* strain DMC.

Primer/probe name	Target	Primer/probe sequence (5′ to 3′)
CM_881F	16S rRNA gene of strain CM	CGCAAGGTTGAAACTCAAAGG
CM_960R		CTTCGCGTTGCTTCGAATTAA
CM_920P		FAM-CAAGCAGTGGATTATG-MGB
Dforq_1205F	16S rRNA genes of *Dehalobacterium formicoaceticum* strain DMC	CACCACGAAAGTTGGCAACA
Dforq_1265R		TTCGGCGACTGCTTCCTT
Dforq_1229P		FAM-AAGTCGATGAGCGAACC-MGB

### Analytical methods

CM, DCM, cDCE, VC, ethene, and methane (CH_4_) were measured by manual headspace injections (0.1 ml) into an Agilent 7890 gas chromatograph (GC) (Santa Clara, CA, USA) equipped with a DB-624 column (60 m length, 0.32 mm i.d., 1.8 μm film thickness) and a flame ionization detector (FID). The GC inlet was maintained at 200°C, the GC oven temperature was kept at 60°C for 2 min, followed by an increase to 200°C at a ramping rate of 25°C min^−1^, and the FID was operated at 300°C as described [39]. The detection limits for DCM, CM, cDCE, VC, ethene, and methane were in the range of 0.01 – 0.08 μmol per 160 ml glass serum bottle.

Acetate and formate were analyzed using an Agilent 1200 series high-performance liquid chromatography (HPLC) system equipped with an Aminex HPX-87H column (Bio-Rad, Hercules, CA, USA) and a UV detector set to 210 nm. The separation occurred at a column temperature of 30°C with isocratic elution (4 mM H_2_SO_4_) at a flow rate of 0.6 ml/min^−1^ for 25 min [49]. Aqueous samples were acidified with 1 M H_2_SO_4_ prior to HPLC analysis. The detection limit for acetate and formate was ~0.5 μmol per bottle (equals to ~0.01 mM).

## Results

### CM fermentation supports reductive dechlorination of cDCE and VC to ethene

**Figure 1:**
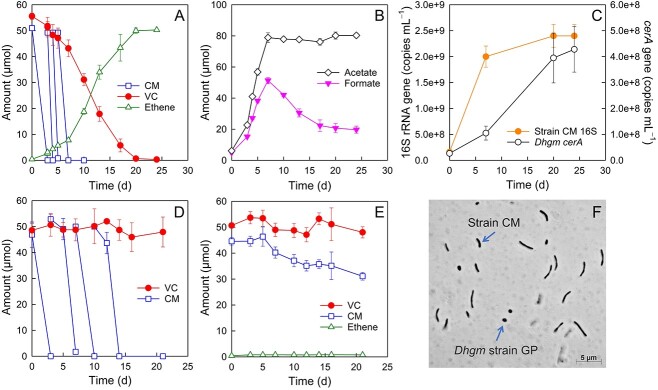
Performance of synthetic strain CM-*Dehalogenimonas* co-cultures amended with CM and VC. (A) CM degradation and reductive dechlorination of VC to ethene in defined medium basal salt medium with CM as the sole source of reducing equivalents. (B) CM fermentation yields acetate and formate, with formate being consumed in cultures during VC reductive dechlorination. (C) Increase of strain CM 16S rRNA gene and *D. etheniformans* strain GP *cerA* gene copies over time. (D) No VC degradation or ethene formation (not shown) occurred in axenic strain CM cultures during growth with CM. (E) Neither VC nor CM was degraded, and no ethene was formed in axenic cultures of *D. etheniformans* (Dhgm) strain GP amended with CM and VC. The data represent the average values of triplicate incubations, and the error bars represent the standard deviations. Error bars are not shown when smaller than the symbol. (F) Phase contrast microscopic image of a CM- and VC-degrading, ethene-producing strain CM and *D. etheniformans* strain GP co-culture.

In co-cultures of the CM degrader strain CM and *D. etheniformans* strain GP, the initial amount of CM (51.0 ± 1.0 μmol) was consumed within 3 days, and three subsequent additions of CM (~200 μmol total) were utilized over a 7-day incubation period (Fig. 1A). Concomitant with CM degradation, formate and acetate formed in the co-cultures, with maximum amounts of 51.4 ± 2.0 μmol of formate and 78.9 ± 3.1 μmol of acetate observed on Day 7 (Fig. 1B). Following CM depletion, formate decreased to 19.7 ± 2.3 μmol, whereas the amount of acetate remained unchanged over a 24-day incubation period (Fig. 1B). The initial amount of VC (55.6 ± 1.2 μmol) was completely reductively dechlorinated to 50.3 ± 0.3 μmol of ethene in the co-cultures after 20 days (Fig. 1A). The number of strain CM cells in the co-cultures, determined by 16S rRNA gene-targeted qPCR, increased from (1.6 ± 0.3) × 10^8^ to (2.4 ± 0.2) × 10^9^ copies per ml, a 15.6-fold increase, during the CM degradation phase (i.e. the initial 7 days of incubation) (Fig. 1C). The number of *D. etheniformans* strain GP cells, determined by qPCR enumeration of the VC reductive dehalogenase gene *cerA*, increased from (2.7 ± 0.7) × 10^7^ to (4.3 ± 0.9) × 10^8^ copies per ml, a 15.9-fold increase, during the phase of VC to ethene reductive dechlorination (Fig. 1C). CM and VC consumption occurred sequentially, with CM fermentation preceding VC reductive dechlorination (Fig. 1A), presumably because CM fermentation generates an electron donor (i.e. formate) and a carbon source (i.e. acetate) *D. etheniformans* strain GP requires for VC reductive dechlorination and growth. The rate of VC reductive dechlorination significantly increased following CM consumption, suggesting CM may have an inhibitory effect on VC reductive dechlorination. In support of this hypothesis, growth experiments with axenic *D. etheniformans* strain GP cultures that received VC, H_2_, and acetate demonstrated that CM at or above the aqueous concentration of ~0.1 mM (~10 μmol per vessel) completely inhibited VC to ethene reductive dechlorination (Supplementary Fig. S1). In axenic cultures of strain CM amended with both CM and VC, only CM was degraded, VC was stable, and no ethene formed (Fig. 1D). In axenic cultures of *D. etheniformans* strain GP amended with CM and VC but without exogenous electron donor (i.e. formate, H_2_) and carbon source (i.e. acetate), no ethene was detected, no growth occurred, and CM and VC were not consumed (Fig. 1E). Consistent with the results of the growth experiments, microscopic analysis visualized both the characteristic curved rod-shaped strain CM and coccus-shaped *Dehalogenimonas* cell morphologies in co-cultures during growth with CM and VC (Fig. 1F).

CM consumption in axenic strain CM cultures resulted in acetate and formate accumulation, indicating that this bacterium cannot metabolize formate (Fig. S2). Strain CM ferments CM plus CO_2_ to both formate and acetate according to (1).


(1) \begin{equation*} 3\ {\mathrm{CH}}_3\mathrm{Cl}+2\ {\mathrm{CO}}_2+2\ {\mathrm{H}}_2\mathrm{O}\to 2\ {\mathrm{CH}}_3{\mathrm{CO}\mathrm{O}}^{\hbox{--} }+{\mathrm{H}\mathrm{COO}}^{\hbox{--} }+6\ {\mathrm{H}}^{+}+3\ {\mathrm{Cl}}^{\hbox{--} } \end{equation*}

Based on (1), strain CM would be expected to produce one-third mol of formate per mol of CM consumed, and the complete degradation of ~200 μmol CM (Fig. 1A) would yield ~66.7 μmol of formate (1), assuming no formate consumption. *D. etheniformans* strain GP in the co-cultures utilized formate as the electron donor to fuel the reductive dechlorination of VC to ethene according to (2).


(2) \begin{equation*} {\mathrm{H}}_2\mathrm{C}=\mathrm{CHCl}+{\mathrm{H}\mathrm{COO}}^{\hbox{--}}\to{\mathrm{H}}_2\mathrm{C}={\mathrm{CH}}_2+{\mathrm{CO}}_2+{\mathrm{Cl}}^{\hbox{--} } \end{equation*}

Based on (2), the amount of formate consumed equals the amount of ethene formed, and 50.3 ± 0.3 μmol of ethene were measured in co-cultures that received ~200 μmol CM. Therefore, a net formation of ~16.4 μmol of formate would be expected based on (1) and (2). A total of 14.8 ± 2.4 μmol of formate was measured in the co-cultures at the end of the incubation period (Fig. 1B), an amount close to the theoretical value. CM fermentation also yields two-thirds mol of acetate according to (1) (i.e. ~133 μmol), and about 80 μmol were measured in the co-cultures that received 200 μmol of CM, consistent with some acetate being utilized as a carbon source for both strains.

**Figure 2:**
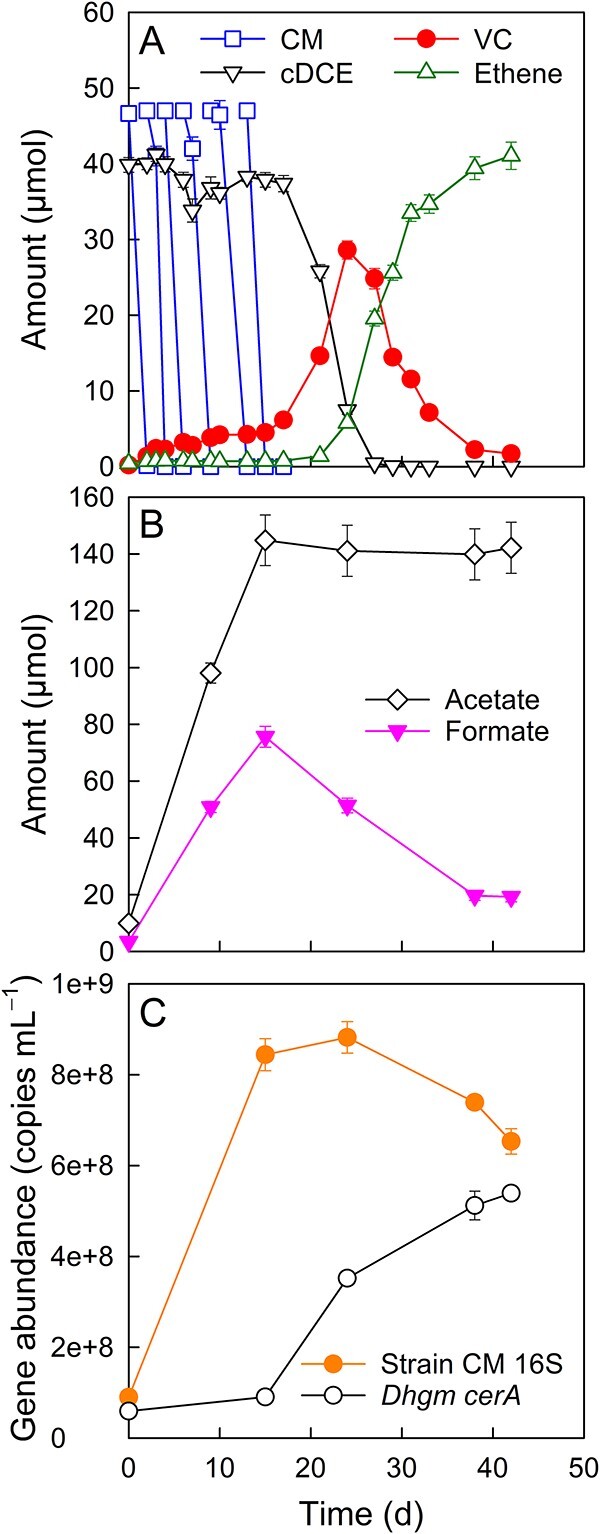
Performance of synthetic strain CM-*Dehalogenimonas* co-cultures amended with CM and cDCE. (A) CM degradation and reductive dechlorination of cDCE to VC and VC to ethene in vessels with CM as the sole source of reducing equivalents. (B) Formation of acetate and formate, the latter being consumed in the observed reductive dechlorination reactions. (C) Increase of strain CM 16S rRNA gene and *D. etheniformans* strain GP *cerA* gene copies in co-cultures amended with CM and cDCE. The data represent the averages of triplicate incubations, and the error bars represent the standard deviations. Error bars are not shown when smaller than the symbol.

Strain CM-*Dehalogenimonas* co-cultures also completely reductively dechlorinated cDCE to ethene, with CM supplied as the sole electron donor (Fig. 2). Approximately 282.0 μmol of CM was completely fermented to formate and acetate over a 15-day incubation period (Fig. 2A) according to (1). A maximum amount of 75.6 ± 3.7 μmol of formate was observed, which decreased to 19.3 ± 1.8 μmol in the co-cultures amended with cDCE (Fig. 2B). Acetate accumulated in the co-cultures amended with cDCE, reaching a maximum amount of 144.9 ± 8.9 μmol (Fig. 2B). cDCE (39.9 ± 1.0 μmol) was reductively dechlorinated to stoichiometric amounts of ethene (41.1 ± 1.8 μmol) with the transient formation of VC (maximum measured amount of 28.6 ± 1.2 μmol) (Fig. 2A). Similar to the observations made in co-cultures that received VC, cDCE to ethene reductive dechlorination occurred at a significantly accelerated rate following CM depletion (i.e. after Day 15), consistent with an inhibitory effect of CM on reductive dechlorination (Supplementary Fig. S1). The number of strain CM cells in the co-cultures increased from (9.0 ± 0.03) × 10^7^ to (8.8 ± 0.3) × 10^8^ per ml, a ~10-fold increase, during the 15-day incubation period (Fig. 2C). Following CM depletion, the growth of strain CM ceased, and the cell numbers decreased slightly to (6.5 ± 0.3) × 10^8^ per ml (Fig. 2C). *D. etheniformans* strain GP cells increased from (5.9 ± 0.02) × 10^7^ to (5.4 ± 0.2) × 10^8^ per ml culture, and this increase mainly occurred after CM depletion and corresponded to the period of cDCE reductive dechlorination to VC and ethene in the co-cultures (i.e. from Day 15 to 42) (Fig. 2C).

According to (1), the fermentation of ~282.0 μmol of CM would yield ~94.0 and 188.0 μmol of formate and acetate, respectively. Based on (3), the reduction of 39.9 ± 1.0 μmol of cDCE to ethene requires about 80.0 μmol of formate as an electron donor.


(3) \begin{equation*} \mathrm{CHCl}=\mathrm{CHCl}+2\ {\mathrm{H}\mathrm{COO}}^{\hbox{--}}\to{\mathrm{H}}_2\mathrm{C}={\mathrm{CH}}_2+2\ {\mathrm{CO}}_2+2\ {\mathrm{Cl}}^{\hbox{--} } \end{equation*}

Therefore, ~14.0 μmol of formate would be expected to remain in co-cultures amended with 282 μmol of CM and 40 μmol of cDCE based on (1) and (3). The measured amount of 15.8 ± 1.8 μmol of formate was close to this theoretical value. CM fermentation would yield 188.0 μmol of acetate, of which 132.2 ± 9.1 μmol was measured at the end of the incubation period, consistent with acetate being utilized as a carbon source by *D. etheniformans* strain GP and strain CM. The experimental data demonstrate that anaerobic CM degradation by strain CM supports organohalide respiration of cDCE and VC to ethene by *D. etheniformans* strain GP in synthetic co-cultures without requiring formate or H_2_ as an exogenous electron donor, or acetate as a carbon source.

### Anaerobic DCM degradation supports VC reductive dechlorination

**Figure 3:**
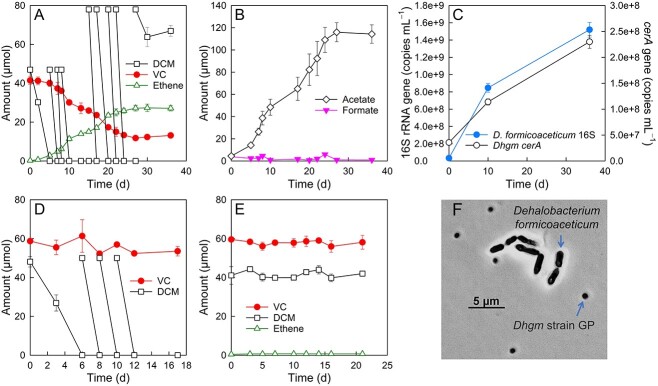
Performance of *Dehalobacterium*-*Dehalogenimonas* co-cultures amended with DCM and VC. (A) DCM degradation and reductive dechlorination of VC to ethene in cultures that received DCM as the sole source of reducing equivalents. (B) Accumulation of acetate and formate concentrations over time. (C) Increase of *Dehalobacterium formicoaceticum* 16S rRNA gene and *D. etheniformans* strain GP *cerA* gene copy numbers in *Dehalobacterium*-*Dehalogenimonas* co-cultures amended with DCM and VC. (D) No VC reductive dechlorination occurred in axenic *Dehalobacterium formicoaceticum* cultures during growth with DCM. (E) VC or DCM were not degraded, and no ethene formation occurred in DCM- and VC-amended axenic *D. etheniformans* strain GP cultures. The data represent the average values of triplicate incubations, and the error bars represent the standard deviations. Error bars are not shown when smaller than the symbol. (F) Phase contrast microscopic image of the *Dehalobacterium*-*Dehalogenimonas* co-culture during growth with DCM and VC. The arrows point at *Dehalobacterium formicoaceticum* strain DMC and *D. etheniformans* (Dhgm) strain GP cells.

Additional cross-feeding experiments tested VC reductive dechlorination in synthetic co-cultures of the DCM fermenter *Dehalobacterium formicoaceticum* strain DMC and *D. etheniformans* strain GP. In *Dehalobacterium*-*Dehalogenimonas* co-cultures, the initial amount of 47.7 ± 1.5 μmol of DCM was consumed within 5 days (Fig. 3A). Additional DCM feedings (~578.0 μmol total) were rapidly consumed, except for the last feeding, and 67.0 ± 2.8 μmol of DCM remained in the incubation vessels (Fig. 3A). VC-to-ethene reductive dechlorination occurred in the *Dehalobacterium*-*Dehalogenimonas* co-cultures; however, the conversion of VC to ethene was slow and incomplete (Fig. 3A). After a 36-day incubation period, VC decreased from 41.4 ± 2.0 to 13.2 ± 0.3 μmol, and the amount of ethene formed (27.1 ± 1.4 μmol) accounted for ~70% of the initial amount of VC (Fig. 3A). *Dehalobacterium formicoaceticum* ferments DCM plus CO_2_ to formate and acetate according to (4) [36, 50].


(4) \begin{equation*} 3\ {\mathrm{CH}}_2{\mathrm{Cl}}_2+4\ {\mathrm{H}}_2\mathrm{O}+{\mathrm{CO}}_2\to 2\ {\mathrm{H}\mathrm{COO}}^{\hbox{--} }+{\mathrm{CH}}_3{\mathrm{CO}\mathrm{O}}^{\hbox{--} }+9\ {\mathrm{H}}^{+}+6\ {\mathrm{Cl}}^{\hbox{--} } \end{equation*}

Based on (4) and the amount of DCM consumed, a theoretical amount of ~170 μmol of acetate would be expected (not considering that some acetate will be consumed as a carbon source). In the experimental systems, a maximum amount of 115.9 ± 8.6 μmol of acetate was measured. Of the theoretical amount of ~340 μmol of formate, no more than 2 μmol (i.e. ~0.04 mM) were analytically captured over the course of the incubation (Fig. 3B). *Dehalobacterium formicoaceticum* strain DMC and *D. etheniformans* strain GP cell numbers increased from (3.4 ± 0.6) × 10^7^ to (1.5 ± 0.1) × 10^9^ copies per ml, and from (3.6 ± 0.1) × 10^7^ to (2.3 ± 0.1) × 10^8^ copies per ml, respectively (Fig. 3C), demonstrating that both bacterial populations grew in the co-cultures amended with DCM and VC. VC was not degraded in axenic *Dehalobacterium formicoaceticum* cultures during growth with DCM (Fig. 3D). Neither DCM nor VC was degraded in axenic *D. etheniformans* strain GP cultures without the exogenous addition of formate or H_2_ as electron donor (Fig. 3E). Microscopic examination of the VC-dechlorinating co-cultures revealed rod-shaped *Dehalobacterium formicoaceticum* strain DMC cells and coccus-shaped *D. etheniformans* strain GP cells in the co-cultures during growth with DCM and VC (Fig. 3F). Like CM, DCM inhibits reductive dechlorination, and the presence of ~0.15 mM DCM (~10 μmol per bottle) completely prevented VC to ethene reductive dechlorination in axenic *D. etheniformans* strain GP cultures (Supplementary Fig. S3).

*Dehalobacterium formicoaceticum* ferments DCM to formate, acetate, and inorganic chloride, whereas `*Ca. Dichloromethanomonas elyunquensis*' present in consortium RM, mineralizes DCM to CO_2_ and H_2_ according to (5) [37].


(5) \begin{equation*} {\mathrm{CH}}_2{\mathrm{Cl}}_2+2\ {\mathrm{H}}_2\mathrm{O}\to 2\ {\mathrm{H}}_2+{\mathrm{CO}}_2+2\ {\mathrm{H}}^{+}+2\ {\mathrm{Cl}}^{\hbox{--} } \end{equation*}

**Figure 4:**
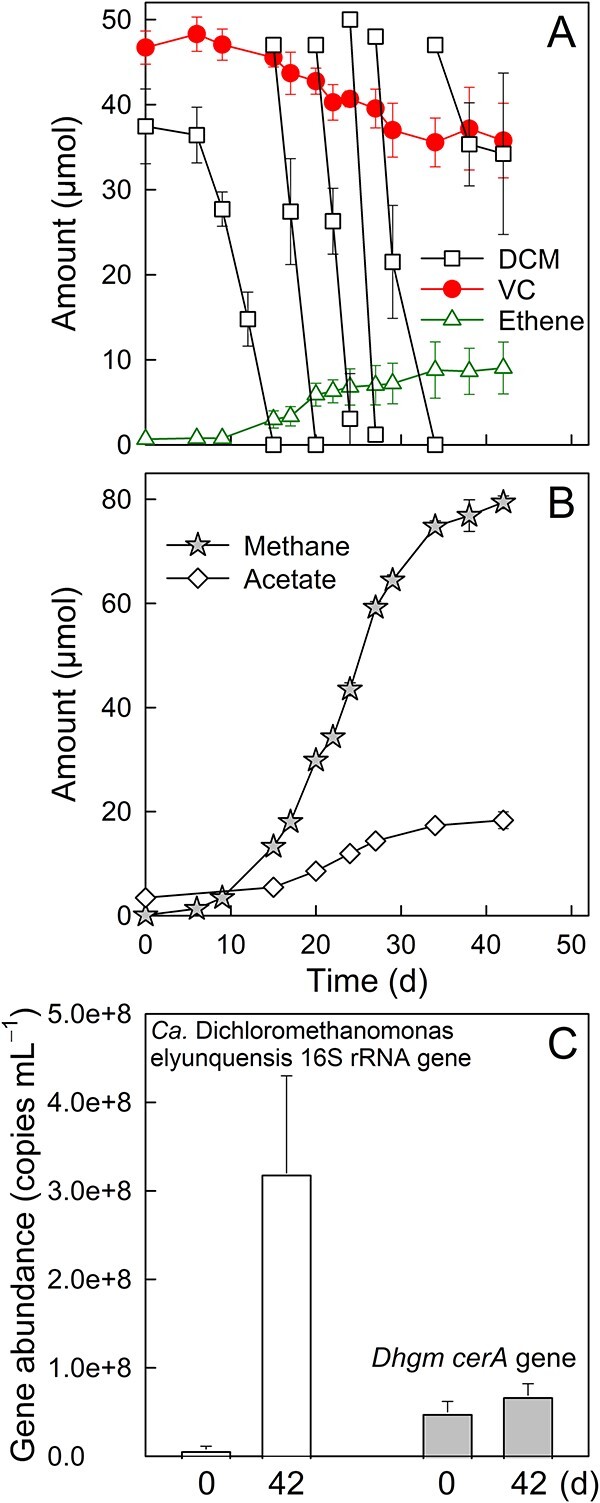
Performance of the DCM-degrading consortium RM inoculated with *D. etheniformans* strain GP and amended with DCM and VC. (A) DCM consumption and reductive dechlorination of VC to ethene in cultures that received DCM as the sole source of reducing equivalents. (B) Accumulation of methane and acetate over time. (C) Increase of `*Ca. Dichloromethanomonas elyunquensis*' 16S rRNA gene and *D. etheniformans (Dhgm) cerA* gene copies in consortium RM inoculated with *D. etheniformans* strain GP and amended with DCM and VC. The data represent the average values of triplicate incubations, and the error bars represent the standard deviations. No error bars are shown when smaller than the symbol size.

During growth with DCM, `*Ca. Dichloromethanomonas elyunquensis*' dominates consortium RM (~50% relative abundance) with hydrogenotrophic methanogens (e.g. *Methanospirillum*) and homoacetogens (e.g. *Acetobacterium*) contributing ~7% and ~2%, respectively [39, 49]. To test if DCM degradation by `*Ca. Dichloromethanomonas elyunquensis*' supports VC reductive dechlorination by *D. etheniformans* strain GP, synthetic mixtures of consortium RM and strain GP (i.e. RM-*Dehalogenimonas* mixtures) were supplied with DCM and VC. Over the course of five repeated DCM feedings, VC decreased from 46.7 ± 2.0 to 35.8 ± 4.4 μmol, and a total amount of 9.0 ± 3.1 μmol of ethene formed, which accounted for around 25% of the initial VC (Fig. 4A). DCM degradation ceased following the last DCM feeding in all replicate cultures. Methane and acetate accumulated in the cultures, reaching amounts of 79.4 ± 1.2 and 18.3 ± 1.6 μmol, respectively (Fig. 4B). Formate was not detected. Based on the stoichiometry of DCM consumption and the formation of methane, acetate, and ethene, around 82.6% of electron equivalents generated during DCM mineralization were recovered in the measured products (Table 2). Consistent with DCM consumption, `*Ca. Dichloromethanomonas elyunquensis*' strain RM cell numbers increased from (8.1 ± 3.2) × 10^6^ to (3.1 ± 1.1) × 10^8^ per ml, and *D. etheniformans* strain GP cell numbers slightly increased from (5.0 ± 1.2) × 10^7^ to (6.9 ± 1.9) × 10^7^ per ml during the 42-day incubation period (Fig. 4C). Neither VC consumption nor ethene formation occurred in DCM-grown RM cultures lacking a strain GP inoculum (data not shown).

**Table 2:** Stoichiometry of substrate consumption, product formation, and calculation of electron recovery in co-cultures of consortium *RM-Dehalogenimonas* mixtures grown with DCM as the sole electron donor as depicted in Fig. 4.^a^

Substrate (μmol bottle^−1^)	Total electrons released (μmol bottle^−1^)	Products [electrons recovered] (μmol bottle^−1^)			Total electron recovery (μmol bottle^−1^)	Electron balance (%)
DCM		Methane	Acetate	Ethene		
242.2 ± 7.1		79.4 ± 1.2	18.3 ± 1.6	9.0 ± 3.1		
	968.9 ± 28.4	[635.2 ± 9.6]	[146.4 ± 12.8]	[18.0 ± 6.2]	800.2 ± 19.2	82.6 ± 2.9

a Electrons available from DCM mineralization were calculated according to (5). The recovered electrons in each product were calculated according to the following equations: (6) CO_2_ + 8 H^+^ + 8 e^−^ → CH_4_ + 2 H_2_O; (7) 2 CO_2_ + 7 H^+^ + 8 e^−^ → CH_3_COO^−^ + 2 H_2_O; and (8) H_2_C=CHCl + H^+^ + 2 e^−^ → H_2_C=CH_2_ + Cl^−^. Electron balance (%) = $\frac{\mathrm{Recovered}\ \mathrm{electrons}}{\mathrm{Released}\ \mathrm{electrons}}\times 100\ \left(\%\right)$. All calculations used data generated from triplicate incubations.

Consortium RM contains hydrogenotrophic acetogens and methanogens, which compete with *D. etheniformans* strain GP for H_2_ as an electron donor in consortium RM-*Dehalogenimonas* mixtures [37–39]. To potentially increase the electron flow toward reductive dechlorination, the methanogenesis inhibitor 2-bromoethanesulfonate (BES, 1 mM) was added. BES completely abolished methanogenesis; however, the addition of BES did not promote the reductive dechlorination of VC to ethene. No ethene was formed, and DCM degradation halted after the second dose of DCM (Supplementary Fig. S4), suggesting that 1 mM BES inhibited DCM mineralization and VC reductive dechlorination.

## Discussion

### Metabolite cross-feeding in anaerobic dechlorinating microbial communities

**Figure 5:**
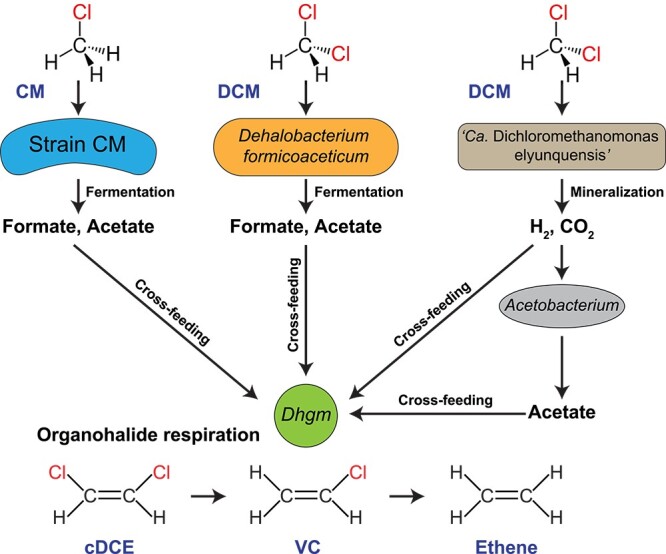
Scheme of metabolite cross-feeding in the strain CM-*Dehalogenimonas* and *Dehalobacterium*-*Dehalogenimonas* co-cultures and consortium RM*-Dehalogenimonas* mixtures. Formate/H_2_ and acetate formed and released during CM and DCM fermentation or mineralization were utilized by *D. etheniformans* strain GP as electron donors and carbon source, respectively, to carry out organohalide respiration with cDCE and VC to ethene.

Obligate OHRB, including strains of the genera *Dehalococcoides* and *Dehalogenimonas*, are metabolic specialists only capable of conserving energy for growth by utilizing organohalogens as terminal electron acceptors [41]. OHRB are broadly distributed in the environment and play important roles for carbon cycling in anoxic ecosystems [51]. *Dehalococcoides* and *Dehalogenimonas* have streamlined genomes with sizes ranging from 1.3 to 2.1 Mb [40, 48, 52], which explains their limited metabolic versatility and dependency on community members for supplying nutrients and growth factors [11, 53–55]. *Dehalococcoides* are restricted to H_2_, while *Dehalogenimonas* can utilize both H_2_ and formate as electron donors [40, 42]. All characterized organohalide-respiring *Dehalococcoidia* are heterotrophs and require external acetate as a carbon source [40, 42]. To date, OHRBs that utilize CM and DCM as electron acceptors have not been found, and other groups of specialized anaerobes utilize these chlorinated C_1_ compounds as energy and carbon sources under anoxic conditions [35–37]. The co-culture experiments demonstrate metabolite cross-feeding between anaerobic CM- or DCM-degrading bacteria and *D. etheniformans*, enabling concomitant degradation of chlorinated methanes and chlorinated ethenes in anoxic environments (Fig. 5).

Metabolite cross-feeding is a widespread phenomenon in anaerobic mixed cultures, including those with dechlorinating capabilities. For example, benzoate fermentation by a *Syntrophus* sp. can support complete reductive dechlorination of tetrachloroethene (PCE) to ethene by organohalide-respiring *Desulfitobacterium* and *Dehalococcoides* populations via interspecies H_2_ cross-feeding [56]. Acetylene (C_2_H_2_) fermentation has been shown to support TCE and VC reductive dechlorination in synthetic co-cultures and in mixed cultures through cross-feeding between acetylene-fermenting acetylenotrophs and OHRB (i.e. *Dehalococcoides mccartyi*) [57, 58]. The complete conversion of chlorobenzene and benzene to CH_4_ and CO_2_ was demonstrated in microcosms bioaugmented with a chlorobenzene-dechlorinating mixed culture and a benzene-degrading methanogenic consortium [59]. The fermentation of benzene generated H_2_ and acetate, which supported chlorobenzene reductive dechlorination to benzene [59]. *Dehalococcoides mccartyi* strain 195 grew more robustly in syntrophic co-cultures with a lactate- or butyrate-fermenting bacterium and a hydrogenotrophic methanogen through metabolite cross-feeding and the elimination of toxic by-products [11, 54, 60].

In the co-cultures, the CM and DCM degraders provided electron donors (i.e. formate and H_2_) and a carbon source (i.e. acetate) to the cDCE- and VC-dechlorinating *Dehalogenimonas* population, which in turn consumed the metabolic products (e.g. H_2_) generated during CM or DCM catabolism. The inhibitory impact of elevated H_2_ on DCM degradation has been demonstrated, and the partnership with H_2_-consuming microorganisms, such as hydrogenotrophic methanogens, homoacetogens, or, as demonstrated in this study, OHRB can alleviate the inhibition [39, 61]. In scenarios like this, a syntrophic partnership benefits both populations through metabolite cross-feeding and represents a mutualistic interaction.

While the strain CM*-Dehalogenimonas* co-cultures completely converted cDCE and VC to stoichiometric amounts of ethene with CM as the sole source of reducing equivalents, VC reductive dechlorination to ethene in DCM-amended *Dehalobacterium-Dehalogenimonas* co-cultures and consortium RM-*Dehalogenimonas* mixtures was slow and incomplete in the batch cutivation experiments. A plausible explanation is formate dehydrogenase (Fdh) activity in *Dehalobacterium formicoaceticum* cells. Consistent with the genomic [47] and biochemical [36] evidence, formate was transiently produced in axenic *Dehalobacterium formicoaceticum* cultures grown with DCM, and acetate accumulated as the end product (Supplemenarty Fig. S5). This consumption of formate explains why repeated DCM feedings were required to provide a sufficient amount of electron donor (i.e. formate) to support VC reduction to ethene in the *Dehalobacterium-Dehalogenimonas* co-cultures. Consortium RM harbors hydrogenotrophic methanogens and homoacetogens, indicating that the competition for electron donors (i.e. H_2_) [37] required continuous DCM feedings to supply sufficient H_2_ for VC reductive dechlorination in the RM-*Dehalogenimonas* mixtures. OHRB consume H_2_ at lower thresholds than H_2_-consuming homoacetogens and methanogens, reflecting the thermodynamics of these terminal electron-accepting processes [62, 63]. Despite the favorable energetics of reductive dechlorination, several studies have demonstrated that hydrogenotrophic processes with faster kinetics (e.g. hydrogenotrophic methanogenesis) can outcompete OHRB for H_2_ in batch culture incubations [64–67]. In agreement with previous studies, only a very small fraction (~2%) of the reducing equivalents generated from DCM mineralization was consumed in VC reductive dechlorination in the consortium RM-*Dehalogenimonas* mixtures, and hydrogenotrophic methanogenesis was the dominant electron-consuming process (Table 2). The methanogenesis inhibitor BES prevented methane formation but did not promote VC reductive dechlorination, presumably because BES can inhibit OHRB [68]. BES may also impact the DCM degrader, as the second DCM dose was not consumed in the RM-*Dehalogenimonas* mixtures amended with BES (Supplementary Fig. S4). Taken together, the slow and incomplete VC reductive dechlorination in DCM-grown co-cultures is largely due to the lack of a sufficient supply of formate or H_2_.

### Implications for bioremediation

Anthropogenic activities have resulted in widespread groundwater contamination, and commingled plumes commonly occur [69], which can impact the efficiency of bioremediation [70–72]. For example, chloroform (CF) is a known potent inhibitor of *Dehalococcoidia* activity, leading to stalled reductive dechlorination of chlorinated ethenes [73–75]. CF can be reductively dechlorinated to DCM by *Dehalobacter* and *Desulfitobacterium* species possessing CF reductive dehalogenases, a conversion regarded as a key step to alleviate CF inhibition of reductive dechlorination [76–79]. Unexpectedly, we observed that CM and DCM inhibit VC reductive dechlorination by *D. etheniformans* strain GP (Supplementary Figs S1 and S3). In addition to electron donor limitation (see above), this inhibition may have also contributed to the slow and incomplete VC-to-ethene reductive dechlorination observed in the DCM-grown co-cultures, and it is possible that *D. etheniformans* strain GP exhibits different susceptibility to CM versus DCM. Future work should determine the CM and DCM inhibitory constants (*K_I_* values) for the reductive dechlorination process. In the co-cultures, the CM and DCM degraders not only provided electron donors (i.e. formate and H_2_) and a carbon source (i.e. acetate) to the cDCE- and VC-dechlorinating *Dehalogenimonas* population but also consumed CM or DCM to alleviate their inhibitory effects on the reductive dechlorination process.

Anaerobic DCM degradation has been shown to supply reducing equivalents for CF reductive dechlorination in a mixed culture, providing evidence for cross-feeding between an anaerobic DCM degrader and a CF-to-DCM respiring *Dehalobacter* strain [80]. A recent study made a similar observation in a mixed culture but, intriguingly, assigned these activities to a single *Dehalobacter* population [81]. Such inter- and intraspecies electron transfer processes have implications for sustainable bioremediation at sites impacted with chlorinated methanes and other organohalogens that can serve as electron acceptors for OHRB. Our co-culture experiments focused on cDCE and VC as electron acceptors, but the findings extend to other organohalogens that OHRB can respire. Thus, this described interdependent process is not limited to environments where chlorinated methanes and chlorinated ethenes co-occur, and CM and DCM catabolism can support reductive dechlorination of other organohalogens.

Conventional enrichment and isolation methodologies have followed the one-contaminant, one-microbe paradigm, and yielded isolates that utilize the pollutant either as an electron donor (e.g. aerobic oxidation of VC) or as an electron acceptor (e.g. organohalide respiration with VC). The bottom-up synthetic ecology experiments using cultures with specific functional properties demonstrated interspecies cooperation and synergisms that resulted in the concomitant degradation of chlorinated C_1_ and C_2_ compounds. The knowledge-based construction of such assemblies (i.e. synthetic ecology) or their direct enrichment from the environment can yield novel consortia, where interspecies cooperation can substantially improve the efficiency of in situ bioremediation.

### Implications for microbial ecology

**Figure 6:**
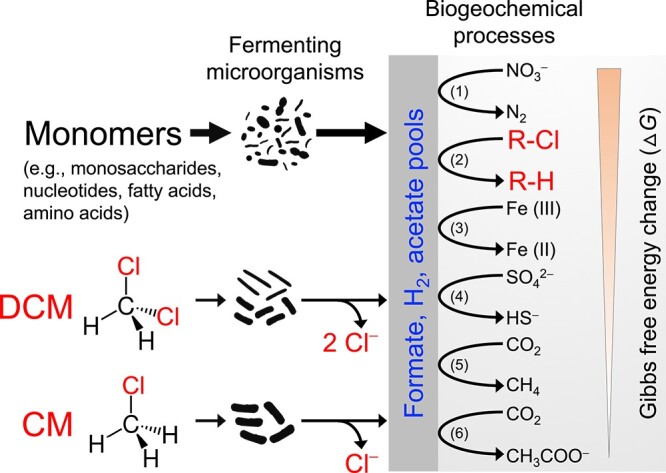
CM and DCM are relevant, yet overlooked, energy sources contributing to the food chains in anoxic environments and support various electron-accepting processes, such as nitrate reduction (1), organohalide respiration (2, demonstrated in this study), ferric iron reduction (3), sulfate reduction (4), methanogenesis (5), and reductive acetogenesis (6).

Both chlorinated methanes and chlorinated ethenes are produced naturally and can coincide in pristine environments [82–84], and it is not surprising that microorganisms have evolved strategies to benefit from these compounds. The degradation of CM and DCM under anoxic conditions [35–37] resembles the catabolism of non-chlorinated C_1_ compounds, such as methanol, methylamines, and methanethiol [85, 86]. Following demethylation, the methyl or methylene group of the C_1_ compounds is channeled into the Wood–Ljungdahl pathway and metabolized via a disproportionation reaction to yield acetate, formate, and H_2_ as products. These products are central intermediates and can fuel other microbial processes through metabolite cross-feeding, such as nitrate reduction, organohalide respiration (demonstrated in this study), ferric iron reduction, sulfate reduction, methanogenesis, and reductive acetogenesis (Fig. 6). The concept of CM or DCM as principle energy sources of anaerobic food webs is exemplified in consortium RM, where DCM catabolism supported methane and acetate formation, and the DCM degrader `*Ca. Dichloromethanomonas elyunquensis*' methanogens, and homoacetogens co-existed over repeated transfers with DCM as the sole substrate for many years [37–39, 87]. Considering that CM and DCM have natural sources in various pristine environments [18–20, 28, 29, 31, 32, 83, 88], these compounds are overlooked, yet potentially relevant substrates for fueling microbial life in energy-deprived anoxic environments, such as groundwater aquifers and the deep subsurface. Other examples include permanently ice-covered, oligotrophic ecosystems in Antarctica, such as Lake Vida. DCM and other chlorinated organohalogens were detected in Lake Vida brine, which presumably formed through abiotic reactions between organic matter and oxychlorines (e.g. perchlorate), and are possible substrates to sustain microbial life in this extreme, oligotrophic environment [88, 89].

The synthetic co-culture experiments demonstrate that anaerobic CM- and DCM-degraders can partner with obligate OHRB. Most organohalogens are part of the natural chlorine cycle, with concentrations of individual chlorinated compounds representing the balance between production and consumption [15, 17, 23, 33, 82–84, 90]. For example, Atlantic Ocean emissions of CM and DCM reaching 500 and 30 ppt (parts per trillion), respectively, have been reported [91]. However, emission data do not inform about fluxes (i.e. the rate of formation minus the rate of consumption), and concentrations of halogenated compounds in pristine environments are compensation concentrations reflecting balanced production and consumption, which are generally too low to be detected with contemporary analytical approaches [32, 92]. Thus, even in environments with low steady-state concentrations of chlorinated compounds, their fluxes may be substantial. Information about the fluxes of organohalogens in natural ecosystems is largely lacking, but the distribution of obligate OHRB and CM-/DCM-degrading bacteria clearly extends beyond contaminated sites where human activities increased the concentrations of chlorinated compounds, emphasizing the relevance of the natural chlorine cycle. Microbial ecologists rarely consider organohalogens as sources of energy and drivers of microbial activity, and our findings highlight that CM and DCM catabolism can enable various electron-accepting processes in anoxic ecosystems. Naturally produced chlorinated methanes are overlooked energy sources in anaerobic food webs and may drive carbon and electron flow in energy-deprived, anoxic environments.

## Supplementary Material

SI_Chen_Cross-feeding_v14_wrae090

## Data Availability

All data generated or analyzed during this study are included in this published article and its supplementary information files.
