# Supplementary material for: Metabolite cross-feeding enables concomitant catabolism of chlorinated methanes and chlorinated ethenes in synthetic microbial assemblies
Source: ISME J. 2024 May 31;18(1):wrae090. doi: 10.1093/ismejo/wrae090 (PMC11170663; doi:10.1093/ismejo/wrae090)
Supplement: SI_Chen_Cross-feeding_v14_wrae090 [file si_chen_cross-feeding_v14_wrae090.pdf]

Supplementary Information

Metabolite cross-feeding enables concomitant catabolism of chlorinated methanes and chlorinated ethenes in synthetic microbial assemblies

Gao Chen[1], Yi Yang[5], Jun Yan[5], and Frank E. Löffler[1-4,#]

[1]Department of Civil and Environmental Engineering, [2]Department of Microbiology, and [3]Department of Biosystems Engineering & Soil Science, University of Tennessee, Knoxville, Tennessee 37996, USA

[4]Biosciences Division, Oak Ridge National Laboratory, Oak Ridge, Tennessee 37831, USA

[5]Key Laboratory of Pollution Ecology and Environmental Engineering, Institute of Applied Ecology, Chinese Academy of Sciences, Shenyang, Liaoning, China, 110016

[#]Corresponding author

Frank E. Löffler

Department of Civil and Environmental Engineering, University of Tennessee, 325 John D. Tickle Engineering Building, 851 Neyland Drive, Knoxville, Tennessee 37996

Phone: (865) 974-4933

E-mail: frank.loeffler@utk.edu

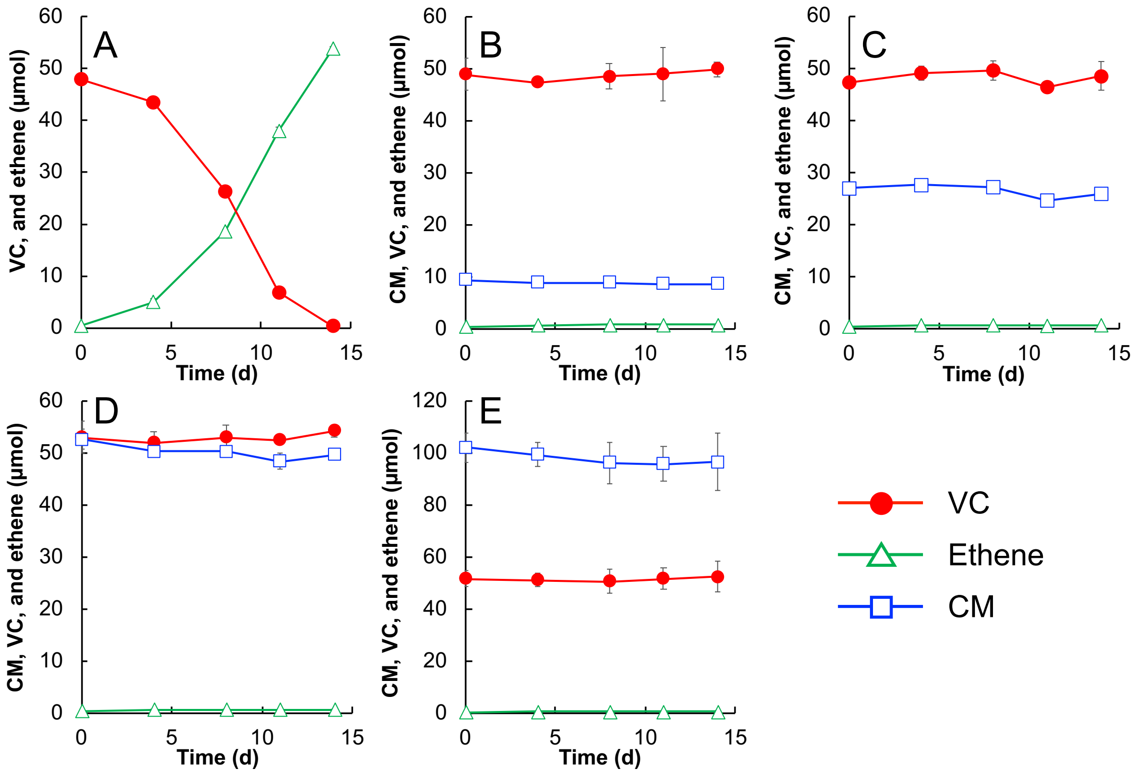

Figure S1. Inhibition of VC to ethene reductive dechlorination by CM in the axenic *Dehalogenimonus etheniformans* strain GP cultures. (A) Without CM, VC was reductively dechlorinated to ethene over a 14-day incubation period. (B-E) In the presence of 10, 30, 50, or 100 µmol of CM per bottle (aqueous concentrations of approximately 0.1, 0.3, 0.5, or 1 mM), neither VC degradation nor ethene formation occurred and CM was not degraded, demonstrating the inhibitory effect of CM on VC to ethene reductive dechlorination in cultures of strain GP. Cultures were incubated in sealed 160-mL glass serum bottles containing 50 mL of basal salt medium. The error bars depict the standard deviation (n = 3) and are not depicted when smaller than the symbol.

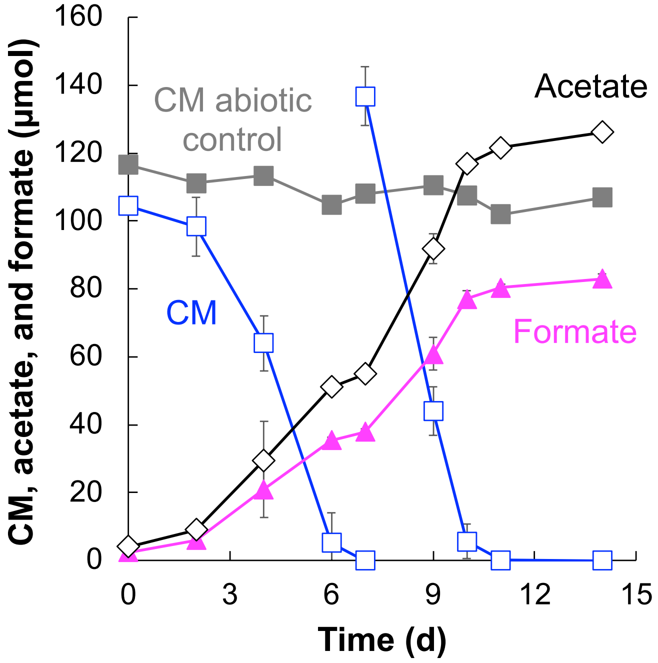

Figure S2. CM fermentation in axenic cultures of strain CM generates formate and acetate as terminal products. Additional CM was spiked at day 7 when the initial feeding of CM had been completely consumed. The grey squares show CM in incubation vessels without inoculum (abiotic control). The error bars depict the standard deviation (n = 3) and are not depicted when smaller than the symbol.

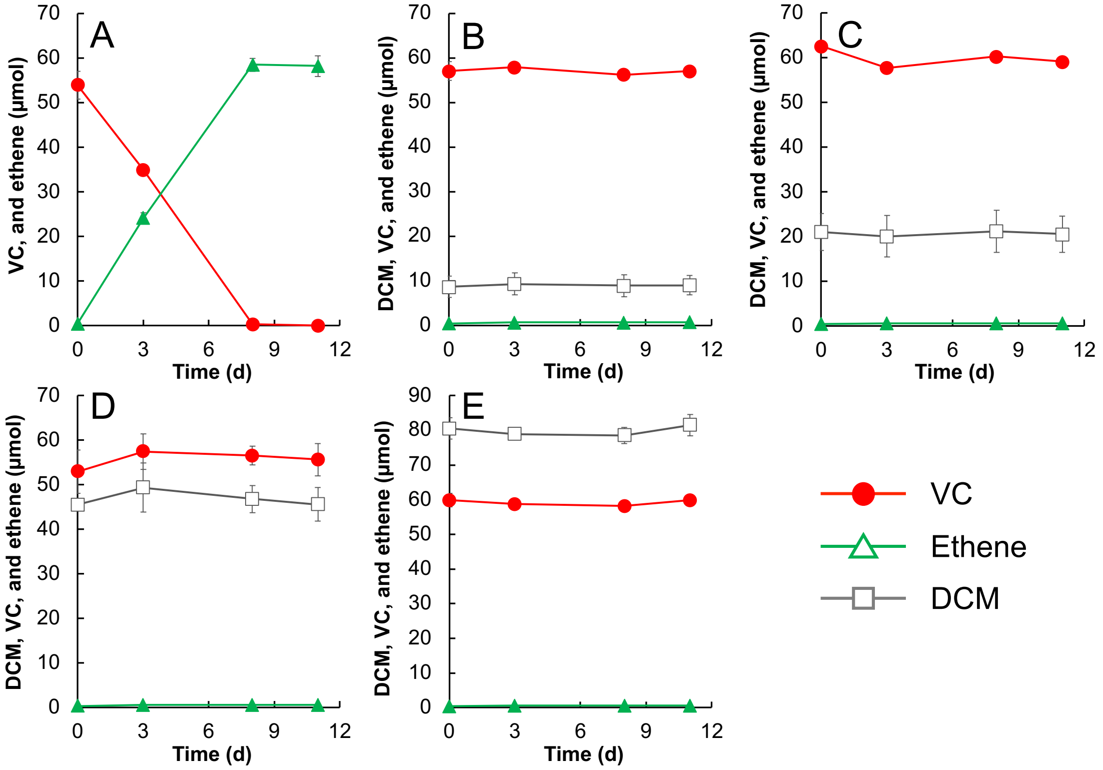

Figure S3. DCM inhibits VC to ethene reductive dechlorination in axenic *Dehalogenimonas etheniformans* strain GP cultures. (A) Without DCM, VC was reductively dechlorinated to ethene over an 8-day incubation period. In the presence of 10, 20, 50, and 80 µmol of DCM per bottle (aqueous concentrations of approximately 0.15, 0.3, 0.75, or 1.2 mM) (B-E), neither VC degradation nor ethene formation occurred in strain GP cultures and DCM was not degraded, demonstrating the inhibitory effect of DCM on VC to ethene reductive dechlorination. The error bars depict the standard deviation (n = 3) and are not depicted when smaller than the symbol.

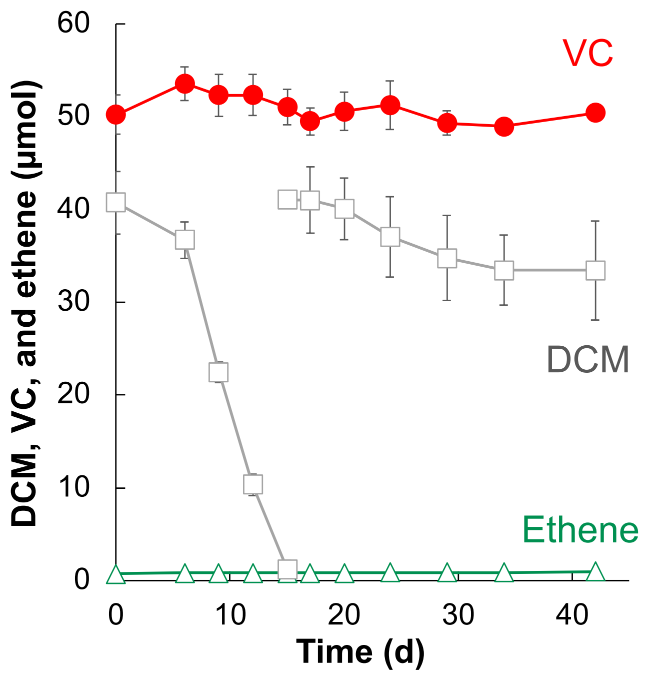

Figure S4. Inhibitory effect 2-bromoethanesulfonate (BES) on DCM degradation and VC to ethene reductive dechlorination in consortium RM inoculated with *Dehalogenimonas etheniformans* strain GP (i.e., *RM-Dehalogenimonas* mixtures). The addition of 1 mM BES completely abolished methane production in the *RM-Dehalogenimonas* mixtures (data not shown). No VC to ethene reductive dechlorination occurred, and DCM degradation ceased after the second feeding in the *RM-Dehalogenimonas* mixtures. The error bars depict the standard deviation (n = 3) and are not depicted when smaller than the symbol.

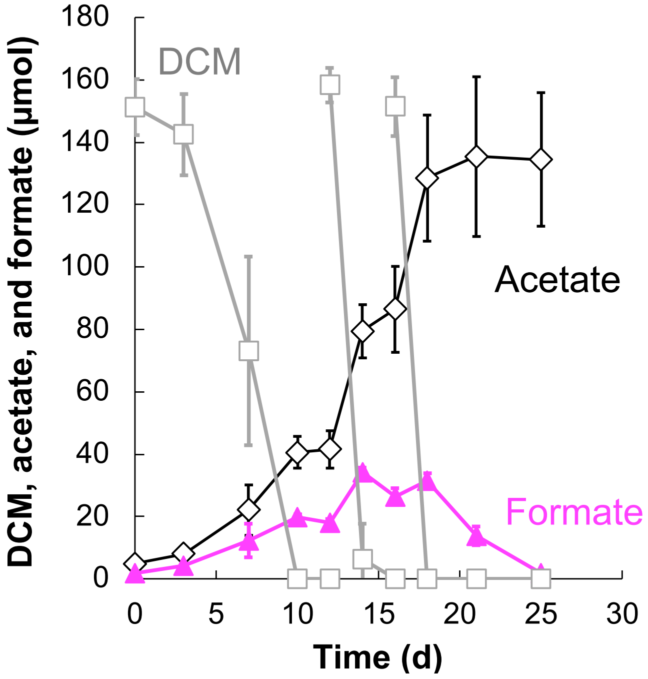

Figure S5. DCM fermentation in axenic *Dehalobacterium formicoaceticum* cultures generates formate and acetate. The bacterium has formate dehydrogenase activity, what explains the consumption of formate over time. Acetate is a terminal product in DCM-grown axenic *Dehalobacterium formicoaceticum* cultures. The error bars depict the standard deviation (n = 3) and are not depicted when smaller than the symbol.
